# Postprandial Insulin and Triglyceride Concentrations Are Suppressed in Response to Breaking Up Prolonged Sitting in Qatari Females

**DOI:** 10.3389/fphys.2019.00706

**Published:** 2019-06-11

**Authors:** Bryna C. R. Chrismas, Lee Taylor, Anissa Cherif, Suzan Sayegh, Nasser Rizk, Abdelrahman El-Gamal, Salwa Hassan Allenjawi, Daniel P. Bailey

**Affiliations:** ^1^Sport Science Program, College of Arts and Science, Qatar University, Doha, Qatar; ^2^Athlete Health and Performance Research Centre, ASPETAR, Qatar Orthopaedic and Sports Medicine Hospital, Doha, Qatar; ^3^School of Sport, Exercise and Health Sciences, Loughborough University, Loughborough, United Kingdom; ^4^Exercise is Medicine, ASPETAR, Qatar Orthopaedic and Sports Medicine Hospital, Doha, Qatar; ^5^Biomedical Sciences, College of Health Sciences, Qatar University, Doha, Qatar; ^6^Department of Medical Imaging, ASPETAR, Qatar Orthopaedic and Sports Medicine Hospital, Doha, Qatar; ^7^School of Sport Science and Physical Activity, Institute for Sport and Physical Activity Research, University of Bedfordshire, Bedford, United Kingdom

**Keywords:** sedentary behavior, cardiometabolic markers, physical inactivity, cardiovascular disease, diabetes

## Abstract

**Background:** Cultural, environmental and logistical factors challenge the Qatari population, particularly females, to engage in physical activity, and there is a high prevalence of diabetes in this population. Sedentary behavior is associated with increased cardiometabolic disease risk and early mortality and breaking up sitting can attenuate postprandial cardiometabolic risk markers. However, no studies have evaluated the cardiometabolic response to breaking up sitting in a Qatari population.

**Purpose:** To examine the effects of breaking up sitting with moderate-intensity walking breaks on cardiometabolic disease markers in Qatari females.

**Methods:** Eleven sedentary (sitting ≥ 7 h/day) females completed two experimental conditions in a cross-over randomized design. The two conditions were identical, except participants either remained seated for 5-h (SIT), or interrupted their sitting every 30-min with a 3-min walk (WALK) on a motorized treadmill (rating of perceived exertion 12–14). A fasting venous blood sample was obtained at baseline (-10-min) followed by samples at 0.5-, 1-, 2-, 3-, 3.5-, 4-, and 5-h. Postprandial cardiometabolic variables (insulin, glucose, triglycerides) were calculated as derivatives of total area under the curve [AUC; total (tAUC), net incremental (iAUC) and positive AUC].

**Results:** Data is reported as effect size; ±90% confidence limit. There was a *most likely* “moderate” lower tAUC (-0.92 ± 0.26), iAUC (-0.96 ± 0.33), and positive AUC (-0.96 ± 0.33) for insulin in WALK compared to SIT. Additionally, there was a *most likely* “moderate” lower tAUC (-0.63 ± 0.37), iAUC (-0.91 ± 0.49), and positive AUC (-0.91 ± 0.49) for triglycerides in WALK compared to SIT. Glucose did not differ between conditions.

**Conclusion:** Breaking up prolonged sitting with moderate-intensity walking offers a culturally compatible intervention to acutely improve cardiometabolic risk markers in sedentary Qatari females. Whilst the data offers promise, the long-term chronic effects of breaking up sitting in Qatari adults requires investigation before population level and/or policy recommendations can be made.

## Introduction

Within Qatar, >70% of the population is either “overweight” or “obese” and 83% participate in little or no physical activity (PA) (Qatar Biobank, 2016). Furthermore, there are concerning levels of clinically high cholesterol (44% prevalence), 13% have impaired glucose tolerance; a strong predictor of future diabetes (Unwin et al., 2002; Bener et al., 2009); and diabetes is prevalent in 17% of Qatari adults (Bener et al., 2009). This may be due to lifestyle behaviors that promote poor regulation of postprandial glucose, insulin and triglycerides, which increase cardiometabolic disease risk (O’Keefe and Bell, 2007). Interventions to improve postprandial metabolism are thus required.

Physical activity levels are low in Qatar with 63% of the population engaging in no recreational PA whatsoever (Qatar National Physical Activity Guidelines, 2014). Particular cultural barriers, beliefs, values and practices, as well as the climate (i.e., hot and humid desert climate), and PA infrastructure, challenge this population to engage with and obtain sufficient PA. Indeed, Islamic traditional clothing (i.e., Abaya and Hijab), adopted widely by Qatari females in public places, has been considered an additional barrier regarding engagement in PA (Klautzer et al., 2014) contributing to 44% of Qatari females achieving <5,000 steps per day (Sayegh et al., 2016). Recent systematic review data shows high levels of physical inactivity within Qatar, which may be due to physical exertion being associated with lower status occupations (Sharara et al., 2018), car-dependent transportation and limited opportunities for PA while at work (Mabry et al., 2016). The yearly direct and indirect costs associated with physical inactivity in Qatar is estimated at $60.7 million (Ding et al., 2016). As part of Qatar’s National Health Strategy (Supreme Council of Health, 2013) and the Nutrition and PA Plan to “*reduce morbidity and mortality attributable to chronic non-communicable diseases*” (Al Thani et al., 2018), Qatar developed its first edition of Qatar’s National PA guidelines (Qatar National Physical Activity Guidelines, 2014). However, these guidelines do not contain any recommendations for reducing or breaking up sedentary time, which could help improve postprandial metabolic risk markers (Healy et al., 2011). Sedentary behavior measurement within the Arab countries (including Qatar) is very limited, with the few studies conducted reporting relatively low levels of daily sedentary time (Mabry et al., 2016). However, these studies used self-report measures which typically underestimate sitting by ≥2 to 3 h/day (Chastin et al., 2014). Given the very low levels of PA in this population, it is highly likely that Qatari females engage in high amounts of sitting that could increase their risk of chronic disease and mortality (Stamatakis et al., 2019). Indeed, the association between sitting and all-cause and cardiovascular-mortality risk is greatest among the least physically active adults (Stamatakis et al., 2019). Cross-sectional evidence has shown that an increased number of breaks in sedentary time is beneficially associated with cardiometabolic risk markers (Healy et al., 2011). Therefore, breaking up sitting time could be a practical and potentially more achievable intervention to improve cardiometabolic risk markers, which can be performed while wearing Islamic clothing. The effects of such an intervention thus requires evaluation in this population.

Several acute laboratory studies have shown improvements in postprandial glucose, insulin, and triglycerides with short-duration (i.e., 2–5-min) and frequent (i.e., every 20–30-min) light or moderate-intensity walking breaks (Peddie et al., 2013; Miyashita et al., 2016; Bailey et al., 2017; Duvivier et al., 2017). However, no study has examined this in a female Arab population. This type of intervention may prove particularly beneficial for Qatari females, who are likely to be highly sedentary (Sayegh et al., 2016), consume a diet that is associated with undesirable postprandial metabolic responses (Al-Thani et al., 2017), and have or are at increased risk of impaired glucose tolerance and diabetes (Bener et al., 2009). Furthermore, females with type 2 diabetes have exhibited a greater attenuation in postprandial glucose in response to breaking up sitting compared to males (Dempsey et al., 2016a), which makes Qatari females an important population to study. Therefore, the aim of this study was to examine the effects of breaking up sitting with short-duration and frequent, moderate-intensity walking breaks on cardiometabolic risk markers in Qatari females. It was hypothesized that glucose, insulin, and triglyceride concentrations would be significantly attenuated in response to breaking up sitting compared within uninterrupted sitting.

## Materials and Methods

### Experimental Design

**FIGURE 1:**
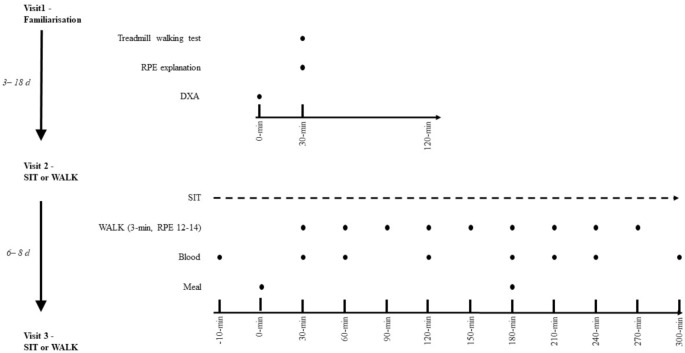
Experimental schematic. SIT, uninterrupted sitting; WALK, breaking up sitting with walking. Both conditions are identical except in SIT, no walking breaks occurred.

This was a randomized crossover design trial. Randomization was completed using GraphPad online QuickCalcs. All experimental procedures were conducted within a temperature controlled laboratory (24 ± 0.3°C). The intervention utilized to break up sitting (see Figure 1 for experimental protocol schematic) has been employed elsewhere (Dunstan et al., 2012; Bailey and Locke, 2015; Bailey et al., 2016). All participants completed a familiarization session followed by two experimental conditions, with temporality indicated on Figure 1. Participants were recruited from February 01, 2017 to November 01, 2017, and were blinded to the condition until arrival on the morning of the first experimental condition. Due to the influence of the female hormonal cycle on glucose metabolism (Bennal and Kerure, 2016), participants completed experimental conditions only in the follicular phase of their menstrual cycle (days 1–10). Participants refrained from exercise for 48-h prior to each condition and recorded volume and timings of all food and liquids consumed in the 24-h prior to the first condition in a food diary and were asked to replicate this intake exactly the day before the subsequent condition. Ethical approval was received from the Anti-Doping Laboratory Qatar Institutional Review Board (IRB# F2016000196). Prior to any experimental procedure occurring, written informed consent was obtained in the spirit of the World Medical Association (2013) and the SPIRIT checklist (Chan et al., 2015).

### Participants

A total of eleven sedentary (sitting ≥ 7 h/day; average 198 MET min/week) Qatari females [median (minimum–maximum) age 27 (21–44) years; height 1.64 (1.57–1.74) meters; body mass 57.8 (47.0–87.4) kg; body fat 36 (24–45) %] completed all experimental procedures. Self-report sitting time was measured using the domain specific sitting time questionnaire (Marshall et al., 2010) and PA (MET min/week) was measured using the International Physical Activity Questionnaire (Craig et al., 2003). Inclusion criteria was premenopausal females aged 18–45 years sitting ≥ 7 h/day. Participants were excluded if they met any of the criteria stated in Table 1. Exclusion criteria was assessed using self-report via completion of an enrolment medical questionnaire. An *a priori* power calculation based on previous research (Bailey and Locke, 2015) estimated that nine participants would be required to detect an effect size of d = 1.47 with an SD of 2.37, a within-person correlation of 0.5, 95% power, and an alpha of 0.05.

**Table 1:** Participant exclusion criteria.

Currently fasting or expecting to fast/modify dietary habits during the time of participation
Self-reported pregnancy
Diagnosed diabetes
Hypertension
Renal failure
Liver disease
History of severe cardiovascular complications
Body mass index > 45 kg/m^2^
Taking glucose or lipid-lowering medication
Smoking
Known physical activity contraindications
Major illness/injury
Other health issues that may limit ability to perform the walking bouts

### Familiarization Visit

Body composition was analyzed using dual-energy X-ray (DXA) absorptiometry (Lunar Idxa, GE Health Care), and stature was measured using a stadiometer (Holtain Ltd., Crymych, Wales). Participants were familiarized, using standardized language, by the same researcher to the Borg 6–20 (“no exertion at all” – “maximum exertion”) rating of perceived exertion (RPE) scale (Borg, 1982) and completed walking on a motorized treadmill (Pulsar, h/p/cosmos, Nussdorf-Traunstein, Germany) at a 1% gradient to determine a self-perceived moderate-intensity speed (RPE 12–14, “somewhat hard”). In order to determine this self-perceived moderate-intensity speed, participants were asked to walk on the treadmill at a speed that corresponded to an RPE 12–14 for 3-min. They were clearly instructed that it must be a walk, and not a run, and were free to adjust the speed of the treadmill until the appropriate RPE was attained. Each minute, participants were asked for their RPE. After a 5-min rest the participants repeated the walking test to confirm the moderate-intensity walking speed. The speed identified was used during the walking breaks in the relevant experimental condition. Treadmill speed was blinded to the participants throughout this test.

### Experimental Conditions

Participants attended the laboratory in the morning (∼08:00 h) following an overnight fast (minimum of 9 h) and were asked to minimize PA during their travel to the laboratory, such as traveling by car. Upon arrival at Aspetar Orthopedic and Sports Medicine Hospital, participants were transported via a wheelchair to the laboratory (∼08:00 h). Participants then transferred to a chair and rested for ∼10 min until the nurse arrived to insert the cannula. Time between arrival and the start of the experimental period was 30–45 min. The two experimental conditions are shown in Figure 1 and were as follows:

### Uninterrupted Sitting (SIT)

During this condition, participants remained seated throughout the 5-h experimental period.

*Sitting interrupted with walking breaks (WALK):* sitting was interrupted every 30-min with 3-min of moderate-intensity walking at the speed that corresponded to an RPE of 12–14 identified during the familiarization session. The mean treadmill belt speed (minimum–maximum) was 6.0 (5.0–8.3) km.h^-1^. The nine walking break bouts resulted in a total of 27-min walking. Frequent walking breaks (i.e., 3-min) performed for a total time of 27-min at a moderate-intensity walking speed was used in line with the Qatar PA guidelines that focus on increasing moderate-intensity PA for at least 30-min per day, five times per week (Qatar National Physical Activity Guidelines, 2014). The frequency of the breaks was based on previous research demonstrating beneficial glucose responses to breaking up sitting every 30-min (Dunstan et al., 2012; Bailey and Locke, 2015; Bailey et al., 2016).

At 0-h a standardized mixed breakfast meal was consumed. A further snack meal was consumed at 3-h to ensure sufficient glucose and insulin stimulus throughout the experimental period. The composition of the breakfast and snack can be seen in Table 2. The food provided are culturally accepted and representative of a typical Qatari diet which is now largely westernized (Al Thani et al., 2018). The breakfast and snack provided 30 and 20%, respectively, of estimated daily energy needs for each participant. Participant energy requirements were calculated using validated equations with a PA factor of 1.4 applied to represent a sedentary day (Mifflin et al., 1990). The glycaemic index of the breakfast and snack was 70 and 67, respectively, which was calculated as described previously (Bailey et al., 2017). Throughout each condition participants were supervised by a researcher to ensure protocol adherence and they were permitted to watched DVDs, read, talk, or work on a laptop when seated (e.g., to simulate a sedentary “office” environment). Participants were asked to minimize excessive movement while seated. During the conditions, participants were able to void when needed and were transported in a wheelchair to the toilets so that they remained inactive. Water was provided *ad libitum* during the first condition and the volume consumed was recorded and then replicated during the subsequent condition.

**Table 2:** Standardized breakfast and snack composition and details. Data is presented as mean ± standard deviation.

Content	Breakfast	Snack
	Cornflakes with whole milk and a croissant	Lusine chocolate puff
Carbohydrate (g)	80 ± 11	35 ± 5
Fat (g)	19 ± 3	14 ± 2
Protein (g)	17 ± 2	6 ± 1
Energy (kcal)	570 ± 78	285 ± 39
Carbohydrate (%)	57	48
Fat (%)	28	44
Protein (%)	15	8

### Blood Collection and Biochemistry

A fasting venous blood sample was obtained at -10-min (baseline), followed by samples at 0.5-, 1-, 2-, 3-, 3.5-, 4-, and 5-h. A cannula (20 GA 1.25 1.1 mm × 32 mm, BD Nexiva, United States) was inserted into the antecubital fossa by a registered and licensed nurse (Qatar Council for Healthcare Practitioners) following standardized procedures. Blood samples were collected into vacutainers in the following order; serum separator vacutainer (4 mL), EDTA (6 mL), and fluoride tube (4 mL) for the determination of triglycerides, insulin and glucose, respectively. Blood samples were centrifuged at 3000-rpm for 10-min (Multifuge^®^ 1S/1S-R). The resulting serum was decanted in triplicate and stored in Eppendorf tubes (Eppendorf, Hamburg, Germany) at -80°C for subsequent analysis. Glucose and triglyceride concentrations were measured using a clinical chemistry analyzer (Medica EasyRA, Medica Corporation, Bedford, MA, United States). Insulin was measured using a commercially available enzyme linked immunoassay kit (Mercodia AB, Uppsala, Sweden, cat# 10-1113-10) with absorbance read using a microplate photometer (Multiskan FC, Thermo Fisher Scientific, Waltham, MA, United States). All samples were run in duplicate, and the intra-assay coefficient of variance was 12.3% for glucose, 4.5% for insulin, and 3.8% for triglycerides.

### Calculation of Outcome Variables

For each cardiometabolic risk marker, total area under the curve (tAUC) was first calculated using the trapezoidal rule. Net incremental area under the curve (iAUC) was calculated by subtracting the baseline area from tAUC, and positive iAUC was calculated whereby any value below baseline was treated as a baseline value. These area under the curve variables were calculated for the total 5-h experimental period in addition to separately for the breakfast and snack postprandial periods. The primary outcome was net iAUC for postprandial glucose across the 5-h experimental period as this is suggested as the most appropriate method for describing postprandial glycaemic responses (Le Floch et al., 1990).

### Statistical Analyses

Statistical analyses were performed using the statistical package for the social sciences (SPSS) version 24 (IBM, SPSS Inc, Chicago, IL, United States) and magnitude-based inferences (MBIs) customizable spreadsheets, using the raw data (Hopkins et al., 2009). Prior to analyses, data were checked and confirmed for assumptions of normality using quantile-quantile (Q-Q) plots (Grafen and Hails, 2002). Descriptive statistics are reported as mean ± standard deviation (SD) and range (minimum–maximum). Differences between conditions (SIT and WALK) for each of the AUC variables (adjusted for baseline blood values, age, and body fat%) were examined using linear mixed models (LMM). Fixed (i.e., condition, baseline values, age, and body fat%) and random (i.e., participants) effects for the LMM were fit for each dependent variable (West et al., 2014). Normality and homogeneity of variance of the residuals were checked using Q-Q plots and scatter plots, respectively, and deemed plausible in each instance. The smallest Hurvich and Tsai criterion (AICC) was used to determine the most appropriate model (Hurvich and Tsai, 1995) in accordance with the principle of parsimony. The least squares mean test provided pairwise comparisons between the conditions. Significant effects were further assessed using Cohen’s d effect sizes (ES), and 90% confidence limits (CLs) using the MBI spreadsheets, and categorized using standardized thresholds of; <0.2 trivial, 0.21–0.60 small, 0.61–1.20 moderate, 1.21–2.0 large, and >2.0 very large (Hopkins et al., 2009) only when the LMM results showed a significant *p* value. A magnitude-based approach was adopted, where differences of >75% likelihood of being greater than the smallest worthwhile effect (0.20 × between subject SD), were reported using the following qualitative descriptions: 75–95% likely, 95–99.5% very likely, and >99.5% most likely (Hopkins et al., 2009). Data is reported as ES; ± 90% CL. Significance was accepted as *p* ≤ 0.05.

## Results

Sixteen participants were recruited for this study. Three participants withdrew from the study prior to the familiarization visit, and two participants withdrew prior to completion of the experimental conditions. The cardiometabolic results for SIT and WALK are shown in Table 3. Baseline (fasting) glucose, insulin and triglycerides did not differ between conditions. Fasting glucose values on average were considered normal (i.e., <6.1 mmol/L) (World Health Organization, 2006) with only one individual presenting a value consistent with impaired fasting glucose. Fasting triglycerides levels for the participants were considered “optimal” or “normal” (Miller et al., 2011). The average body fat in the present study was 36% [minimum–maximum (24–45 %)] suggesting that overall the sample was obese; only *n* = 2 participants were considered “non-obese.”

**Table 3:** Cardiometabolic risk marker values in SIT and WALK. Data are presented as median (minimum–maximum).

	SIT	WALK
Fasting glucose (mmol/L)	5.0 (3.9–6.2)	5.1 (3.8–5.9)
Fasting insulin (μU/mL)	7.7 (4.2–8.9)	7.3 (5.0–11.9)
Fasting TG (mmol/L)	0.8 (0.6–1.0)	0.8 (0.6–1.1)
tAUC glucose (mmol/L.5-h)	29.5 (23.4–34.2)	25.6 (18.5–38.1)
Net iAUC glucose (mmol/L.5-h)	3.2 (-5.7–13.0)	0.30 (-2.2–12.2)
Positive iAUC glucose (mmol/L.5-h)	3.2 (0.7–13.4)	2.0 (0.1–12.6)
tAUC insulin (μU/mL.5-h)	86.1 (174.5–421.6)	70.8 (113.0–294.7)^∗^
Net iAUC insulin (μU/mL.5-h)	82.6 (453.6–381.4)	65.9 (68.4–251.3)^∗^
Positive iAUC insulin (μU/mL.5-h)	82.6 (153.6–382.8)	59.5 (68.4–250.9)^∗^
tAUC TG (mmol/L.5-h)	1.0 (3.2–6.6)	1.2 (2.6–6.4)^∗^
Net iAUC TG (mmol/L.5-h)	0.8 (0.4–2.8)	0.5 (-0.3–1.6)^∗^
Positive iAUC TG (mmol/L.5-h)	1.1 (0.4–2.8)	0.7 (0.0–5.5)^∗^

*^∗^ significant difference between WALK and SIT (p < 0.01); SIT, uninterrupted sitting; WALK, breaking up sitting with walking; TG, triglycerides; tAUC, total area under the curve; iAUC, incremental area under the curve*.

**FIGURE 2:**
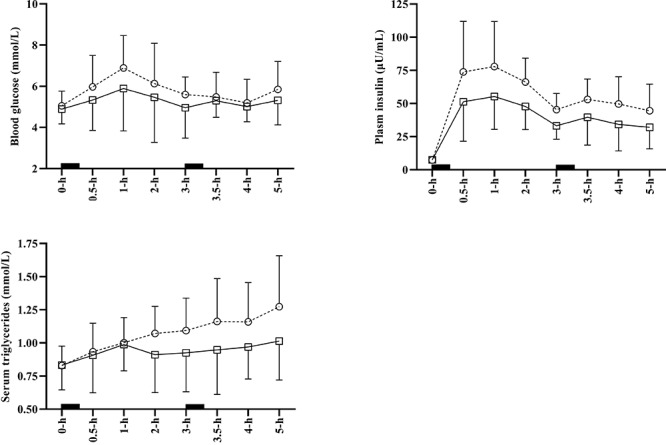
Average time course data for glucose, insulin and triglycerides for SIT (open circles and dashed line), and WALK (open squares and sold line). Error bars indicate standard deviation and solid black squares indicate meal consumption. Some error bars have been omitted to aid clarity. SIT, uninterrupted sitting; WALK, breaking up sitting with walking.

For the total 5-h experimental period, there was a significant main effect of condition for insulin tAUC (*p* < 0.001), iAUC (*p* < 0.001), and positive iAUC (*p* < 0.001). Specifically, there was a *most likely* “moderate” lower tAUC (-0.92 ± 0.26), iAUC (-0.96 ± 0.33), and positive iAUC (-0.96 ± 0.33) for insulin in WALK compared to SIT. Additionally, there was a significant main effect of condition for triglyceride tAUC (*p* = 0.009), iAUC (*p* = 0.005), and positive iAUC (*p* = 0.009). Compared with SIT, there was a *most likely* “moderate” lower tAUC (-0.63 ± 0.37), iAUC (-0.91 ± 0.49), and positive iAUC (-0.91 ± 0.49) for triglycerides during WALK. Glucose tAUC, iAUC and positive iAUC did not differ significantly between conditions (*p* ≥ 0.14). Time course data for changes in glucose, insulin and triglycerides is shown in Figure 2.

When evaluating the breakfast postprandial period, the findings were consistent with the total 5-h experimental period with none of the glucose AUC variables being different between conditions (all *p* > 0.220; see Supplementary Table 1). There was a significant main effect of condition for insulin tAUC (*p* = 0.047), iAUC (*p* = 0.001), and positive iAUC (*p* = 0.004). Specifically, there was a *most likely* “moderate” lower tAUC (-0.89; ± 0.39) and iAUC (-0.93; ± 0.41), and a *very likely* “moderate” lower positive iAUC in WALK compared to SIT. For triglycerides, there was a significant main effect of condition for tAUC (*p* = 0.049), and iAUC (*p* = 0.007). Within WALK there was a *likely* “small” lower tAUC (-0.42 ± 0.39) and *very likely* “moderate” lower iAUC (-0.82 ± 0.47) compared to SIT. For the snack postprandial period, there was a significant main effect of condition for insulin tAUC (*p* = 0.017), and triglyceride tAUC (*p* = 0.031). Within WALK there was a *very likely* “moderate” lower insulin tAUC (-0.80 ± 0.54) and *likely* “moderate” lower triglyceride tAUC (-0.69 ± 0.51) compared with SIT (see Supplementary Table 2). There were no significant differences for the snack postprandial period for any other AUC variables (*p* > 0.33).

## Discussion

The main findings in the present study were that breaking up sitting with 3-min of moderate-intensity walking every 30-min improved postprandial triglycerides and insulin over a 5-h period compared to uninterrupted sitting.

In comparison to findings in the present study, previous acute studies examining the effects of regularly breaking up sitting time on triglycerides have shown mixed results (Altenburg et al., 2013; Peddie et al., 2013; Bailey and Locke, 2015; Miyashita et al., 2016; Maylor et al., 2018). In agreement with the present findings, previous research has found that interrupted sitting every 30 min with brisk walking significantly reduced and triglyceride concentrations (Miyashita et al., 2016). It appears from previous studies that frequent short light or moderate-intensity PA breaks (Peddie et al., 2013; Miyashita et al., 2016), rather than longer duration less frequent PA breaks (Altenburg et al., 2013; Maylor et al., 2018), may be superior relative to improving postprandial triglycerides. These frequency dependent responses may be a result of enhanced lipoprotein lipase enzyme activity due to greater stimuli (e.g., more frequent and higher volume of activity bouts) (Peddie et al., 2013). The present study supports the efficacy of frequent moderate-intensity PA breaks in for suppressing postprandial triglycerides in Qatari females. However, studies that directly compare different frequencies and intensities of PA breaks are needed to confirm a dose-response effect. As hypertriglyceridemia (elevated postprandial triglycerides) significantly increases the risk of cardiovascular disease (O’Keefe and Bell, 2007; Watson and Wiesner, 2016), breaking up sitting in Qatari adults could be an important public health target given the concerning high prevalence of cardiometabolic disease in this population (Qatar Biobank, 2016). Long-term intervention studies are thus required to establish the potential chronic effects of breaking up siting time.

The postprandial glucose and insulin response to breaking up sitting is mixed within the literature (Dunstan et al., 2012; Peddie et al., 2013; Bailey et al., 2016, 2017; Hansen et al., 2016; Miyashita et al., 2016). In agreement with the present study, short-duration and frequent walking breaks have been shown to attenuate insulin concentrations (Dunstan et al., 2012; Peddie et al., 2013). Conversely, no effect on insulin concentration has also been reported in response to breaking up sitting with light or moderate-intensity walking (Bailey et al., 2016, 2017; Hansen et al., 2016; Miyashita et al., 2016). However, it should be noted that none of these studies were powered to detect changes in postprandial insulin and this may thus lead to null findings. Also in contradiction to findings in the present study, reduced glucose concentrations have been reported in response to moderate-intensity walking breaks (Dunstan et al., 2012; Peddie et al., 2013; Miyashita et al., 2016; Bailey et al., 2017). Variation in the findings from these studies may be due to composition of the test meals provided (i.e., carbohydrate content, glycaemic index, and load), individual differences (i.e., genetics), and duration and intensity of PA used to break up sitting. Furthermore, the cardiometabolic health profile of the participants studied (e.g., normal vs. impaired glycaemia) may affect glucose and insulin responses to PA breaks (Benatti and Ried-Larsen, 2015). Although the body fat % of the participants in the present study was relatively high (i.e., >30%), only one participant reached the criteria for impaired fasting glucose levels. Therefore, overall the participants in the present study can be categorized as overweight but metabolically healthy. Studies in people with high adiposity (Dunstan et al., 2012), impaired glucose levels (Henson et al., 2016), and Type 2 diabetes (Dempsey et al., 2016a) have reported greater attenuation of postprandial insulin and glucose in response to breaking up sitting (Dempsey et al., 2016a) compared with studies in “healthy” samples (Bailey and Locke, 2015; Bailey et al., 2016; Maylor et al., 2018). Individuals with high body fat who are metabolically impaired may thus benefit more from breaking up sitting time than those who are healthy and non-overweight. However, further studies adequately powered to detect the interacting effects of health status with breaking up sitting are required before definitive conclusions can be made in this respect.

In the present sample, it appears that breaking up sitting enhances insulin-stimulated glucose disposal in light of the unchanged postprandial glucose levels despite a reduction in insulin concentrations. It has been postulated that breaking up sitting may improve insulin sensitivity via upregulation of insulin signaling pathways (e.g., the Akt-mediated insulin-sensitive glucose uptake pathway) (Bergouignan et al., 2016), increases in muscle insulin sensitivity, or changes in sympathetic nervous system activity (Dempsey et al., 2016b). However, it is not possible to determine which of these potential mechanisms are responsible for the reductions in postprandial insulin in the present study and future research should evaluate such mechanisms to elucidate the reasons for cardiometabolic benefits observed in response to breaking up sitting.

The present data and associated discussion above centers on acute studies, and evidently longer term interventions produce more robust evidence, from which PA policy adoption evolves. Currently, such longer-term evidence from experimental designs using a comparative intervention and outcome measures to the present study is not available. As aforementioned, >70% of the Qatari population is either “overweight” or “obese” (Qatar Biobank, 2016). Furthermore, 86% of Qatari females report engaging in no recreational PA whatsoever (Qatar National Physical Activity Guidelines, 2014) The participants in the current study thus appear to represent the general female Qatari population and future research evaluating the long-term chronic response to breaking up sitting should be conducted in order to corroborate the findings from this acute study and establish evidence based guidelines for reducing sedentary behavior in this population.

This study is limited as it was a laboratory controlled study, and therefore, results are not generalisable to a workplace or domestic environment. Light-intensity walking (as opposed to the moderate-intensity walking employed) could have greater ecologically validity. Future work should examine the effects of light-intensity walking breaks on cardiometabolic responses in Qatari females within the workplace and during leisure time.

## Conclusion

Breaking up prolonged sitting with short-duration frequent moderate-intensity walking improves postprandial triglyceride and insulin responses in Qatari females. Given the low PA levels and undesirable diet (despite country specific recommendations for both), this population predisposes itself to a high risk of cardiometabolic disease and early mortality. Therefore, breaking up sitting time, which may be a more achievable target than increasing moderate-to-vigorous PA levels, could be recommended for reducing cardiometabolic disease risk in this population. However, before population wide recommendations or policy progression is initiated, it is important to examine the long-term feasibility and efficacy of breaking up sitting in the Qatari population.

## Ethics Statement

Ethical approval was received from the Anti-Doping Laboratory Qatar Institutional Review Board (IRB# F2016000196). Prior to any experimental procedure occurring, written informed consent was obtained in the spirit of the World Medical Association (2013) and the SPIRIT checklist.

## Author Contributions

BC, DB, and LT contributed to study conception, interpretation, and manuscript writing. BC, AC, SS, NR, AE-G, and SA contributed to data collection, participant recruitment, and analyses. All authors edited and approved the final manuscript.

## Conflict of Interest Statement

The authors declare that the research was conducted in the absence of any commercial or financial relationships that could be construed as a potential conflict of interest. The handling Editor declared a past co-authorship with authors BC and LT.
