# Supplementary material for: Postprandial Insulin and Triglyceride Concentrations Are Suppressed in Response to Breaking Up Prolonged Sitting in Qatari Females
Source: Front Physiol. 2019 Jun 11;10:706. doi: 10.3389/fphys.2019.00706 (PMC6579923; doi:10.3389/fphys.2019.00706)
Supplement: Supplementary file 1 [file Table_1.DOCX]

Supplementary Table 1. Cardiometabolic risk marker values in SIT and WALK for the breakfast postprandial period only. Data are presented as median (minimum – maximum).

|  | SIT | WALK |
| --- | --- | --- |
| tAUC glucose (mmol/L.3-h) | 17.6 (14.9 – 23.5) | 15.6 (10.1 – 27.5) |
| Net iAUC glucose (mmol/L.3-h) | 2.3 (-1.6 – 11.8) | 0.5 (-2.7 – 12.0) |
| Positive iAUC glucose (mmol/L.3-h) | 2.3 (0.5 – 12.3) | 1.4 (0.0 – 12.0) |
| tAUC insulin (μU/mL.3-h) | 171.9 (95.7 – 277.2) | 126.6 (75.0 – 209.5)* |
| Net iAUC insulin (μU/mL.3-h) | 151.2 (83.2 – 252.4) | 106.7 (48.3 – 183.2)* |
| Positive iAUC insulin (μU/mL.3-h) | 151.2 (83.2 – 252.4) | 100.5 (48.3 – 183.2)* |
| tAUC TG (mmol/L.3-h) | 3.2 (1.9 – 3.6) | 2.9 (1.5 – 3.6)* |
| Net iAUC TG (mmol/L.3-h) | 0.5 (0.2 – 1.1) | 0.2 (-0.2 – 1.0)* |
| Positive iAUC TG (mmol/L.3-h) | 0.5 (0.2 – 1.1) | 0.35 (0.0 – 5.2) |

* significant difference between WALK and SIT (p < 0.05); SIT = uninterrupted sitting; WALK = breaking up sitting with walking; TG = triglycerides; tAUC = total area under the curve; iAUC = incremental area under the curve
