# Supplementary material for: Postprandial Insulin and Triglyceride Concentrations Are Suppressed in Response to Breaking Up Prolonged Sitting in Qatari Females
Source: Front Physiol. 2019 Jun 11;10:706. doi: 10.3389/fphys.2019.00706 (PMC6579923; doi:10.3389/fphys.2019.00706)
Supplement: Supplementary file 2 [file Table_2.DOCX]

Supplementary Table 2. Cardiometabolic risk marker values in SIT and WALK for the snack postprandial period only. Data are presented as median (minimum – maximum).

|  | SIT | WALK |
| --- | --- | --- |
| tAUC glucose (mmol/L.1.5-h) | 10.6 (7.6 – 13.7) | 10.3 (8.5 – 13.2) |
| Net iAUC glucose (mmol/L.1.5-h) | -0.2 (-2.1 – 1.6) | 0.2 (-4.0 – 4.7) |
| Positive iAUC glucose (mmol/L.1.5-h) | 0.2 (0.0 – 1.6) | 0.5 (0.0 – 4.7) |
| tAUC insulin (μU/mL.1.5-h) | 89.7 (58.5 – 154.3) | 63.3 (37.8 – 124.7)* |
| Net iAUC insulin (μU/mL.1.5-h) | 4.9 (-33.5 – 42.8) | -6.4 (-26.2 – 65.2) |
| Positive iAUC insulin (μU/mL.1.5-h) | 8.7 (0 – 42.8) | 1.75 (0.0 – 65.2) |
| tAUC TG (mmol/L.1.5-h) | 2.3 (1.3 – 3.4) | 1.9 (1.0 – 2.8)* |
| Net iAUC TG (mmol/L.1.5-h) | 0.1 (-0.1 – 0.6) | 0.0 (-0.2 – 0.7) |
| Positive iAUC TG (mmol/L.1.5-h) | 0.2 (0.0 – 0.6) | 0.1 (0.0 – 2.4) |

* significant difference between WALK and SIT (p < 0.05); SIT = uninterrupted sitting; WALK = breaking up sitting with walking; TG = triglycerides; tAUC = total area under the curve; iAUC = incremental area under the curve
